# The previously undescribed variant of the thyrohyoid muscle and its potential impact on surgical procedures

**DOI:** 10.1007/s00276-024-03388-9

**Published:** 2024-06-14

**Authors:** Krystian Maślanka, Nicol Zielinska, Piotr Karauda, Andrzej Węgiel, Małgorzata Niemiec, Łukasz Olewnik

**Affiliations:** 1 Department of Anatomical Dissection and Donation, Medical University of Lodz, Lodz, Poland; 2 First Department of Cardiology, School of Medicine in Katowice, Medical University of Silesia, Katowice, Poland; 3 Department of Clinical Anatomy, Masovian Academy in Płock, Płock, Poland

**Keywords:** Infrahyoid muscle, Thyrohyoid, Cricohyoid, Cricothyrohyoid, Infrahyoid flap, Prelaryngeal muscle, Prelaryngeal surgery, Reconstructive surgery, Plastic surgery

## Abstract

The thyrohyoid muscle belongs to the infrahyoid group located in the carotid triangle. It normally originates from thyroid cartilage and inserts into hyoid bone. Quite often, it is continuous with the sternohyoid muscle. Furthermore, there are variants that have their origin in the cricoid cartilage only, however, this occurs very rarely. During anatomical dissection, a two-headed variant of this muscle was found. One head had its origin in the cricoid cartilage and the other in the thyroid cartilage. This variant of thyrohyoid had not been previously described in the available literature. Therefore, we believe that it may be referred to as the cricothyrohyoid muscle. As the thyrohyoideus is often used as a landmark during surgical procedures in the prelaryngeal area and as a muscle graft, a thorough knowledge of its anatomy and variation is extremely important. We speculate that the two-headed version of this muscle may be problematic during surgical procedures in this region, however, it may also provide more options as a muscular graft.

## Introduction

The thyrohyoideus (TH) is a quadrilateral infrahyoid muscle located deep to the sternohyoid (SH) and the superior belly of the omohyoid muscle (OM) on the lateral aspect of the thyroid lamina in the carotid triangle [14]. TH usually originates from the oblique line of thyroid cartilage and is inserted into the greater horn of the hyoid bone (HB) [25]. It may also originate from cricoid cartilage, in which case it is described as cricohyoid muscle—a variant of the thyrohyoideus [1]. Moreover, TH frequently is continuous with SH [1].

TH is usually supplied by the hyoid branch of the superior thyroid artery and rarely by the lingual and superior laryngeal vessels [32]. Its innervation originates from the ventral ramus of C1 of the cervical plexus, and as a branch, it courses along with the hypoglossal nerve [4, 30]. It is the only structure of the infrahyoid group that is not innervated by a nerve that originates in the ansa cervicalis [17]. The ansa cervicalis is the regular nerve donor in recurrent laryngeal nerve injury, however, it is not always possible to use it [7]. Some studies report that in such cases, a nerve branch for the TH may be an alternative [4, 7].

The main functions of this muscle involve elevating the larynx and lowering HB [19]. During phonation, the TH has the highest activity of the infrahyoid group [22]. It is one of the muscles that reallocates power to the cricothyroid complex from suprahyoid muscles, which consequently facilitate the opening of the esophageal sphincter [12].

The TH, including its nerve, is taken as a graft, usually with the other muscles from the infrahyoid group, to restore the medium defects, e.g., in the larynx, throat and esophagus [6, 20, 31].

There is not much information about the variability of this muscle in the available literature.

## Case report

An 83-year-old body donor was admitted to the Department of Anatomical Dissection and Donation, Medical University of Lodz, Poland, for didactic and scientific purposes. During the standard anatomical dissection of the neck region, we discovered a previously unreported variant of the thyrohyoid muscle. The further steps of the proceedings included detailed characterization, measurements and photo documentation.

**Fig. 1:**
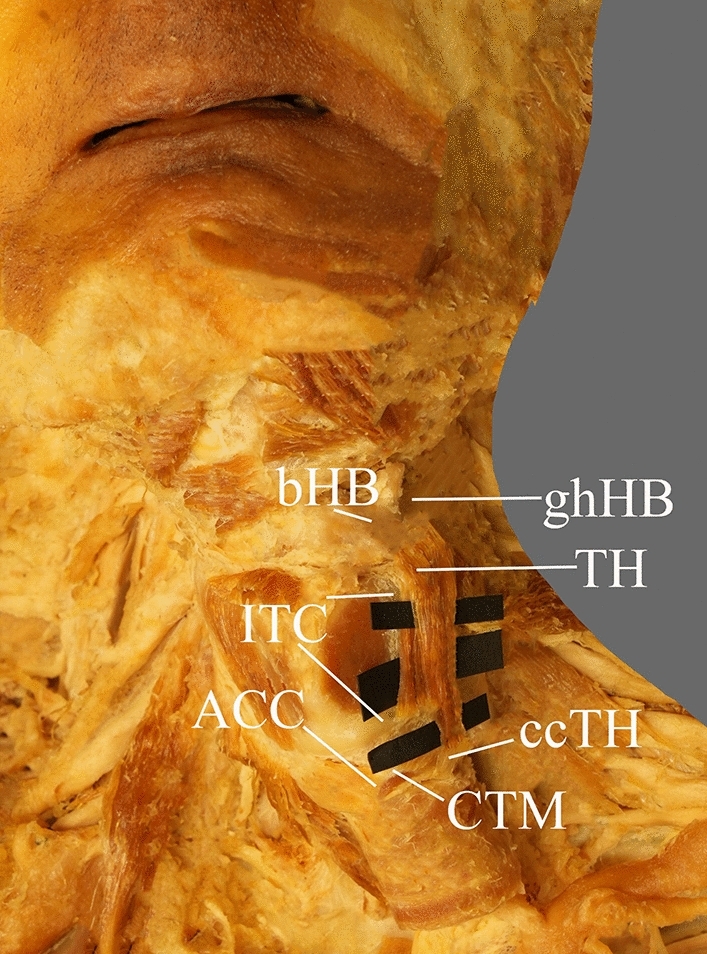
Anterolateral region of the neck. The two-headed thyrohyoid, with each of the heads originating separately from the cricoid cartilage and thyroid cartilage. *TH* thyrohyoid muscle, *ccTH* attachment to the cricoid cartilage of the thyrohyoid muscle, *lTC* lamina of the thyroid cartilage, *ACC* arch of the cricoid cartilage, *CTM* cricothyroid muscle, *bHB* body of the hyoid bone, *ghHB* greater horn of the hyoid bone,

**Fig. 2:**
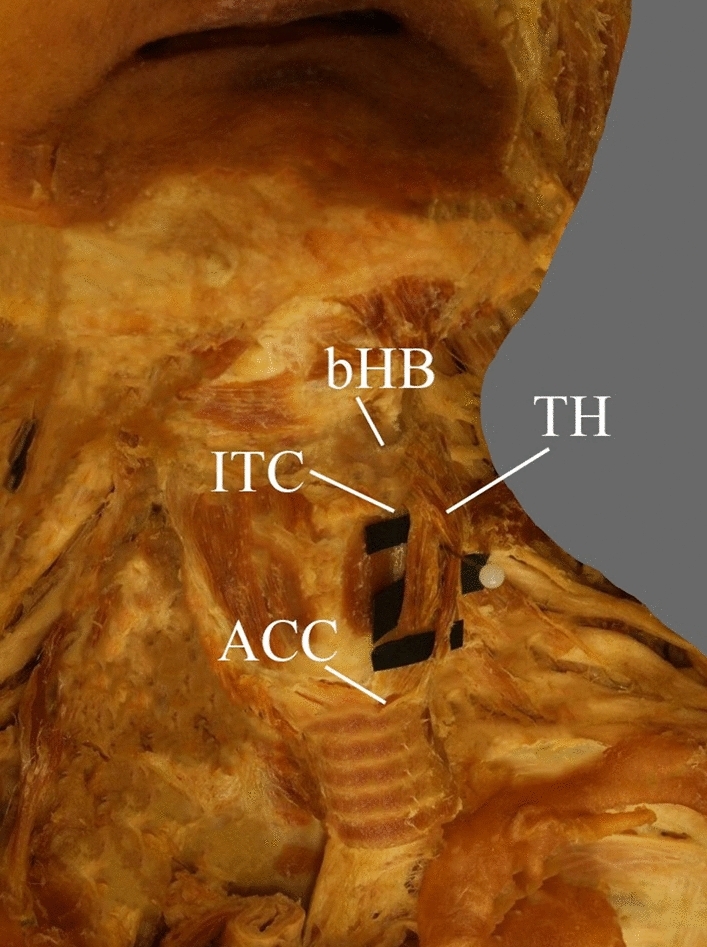
Anterolateral region of the neck. The two-headed thyrohyoid. *TH* thyrohyoid muscle, *lTC* lamina of the thyroid cartilage, *ACC* arch of the cricoid cartilage, *bHB* body of the hyoid bone

We noticed a two-headed TH, with each of the heads originating separately from the thyroid cartilage and cricoid cartilage, respectively—Fig. 1. The two heads were fused above the attachment to the greater horn of the HB—Fig. 2. The medially located head was attached to the oblique line and inferior tubercle of the thyroid cartilage, while the lateral head was attached to the anterolateral aspect of the cricoid cartilage. Consequently, the medial head was shorter, wider, and thicker than the lateral head—its dimensions are shown in Table 1. Furthermore, the lateral head, around its proximal attachment, was connected to the cricothyroid muscle by a few thin muscle fibers.

**Table 1:** Morphometric measurements of the thyrohyoid muscle and its nerve branch [mm]

Attachment point		Width* (mm)	Thickness* (mm)	Length (mm)
First belly	Cricoid cartilage	9	0.8	50.9
Second belly	Thyroid cartilage**	11	0.7	36.1
Proximal attachment of the TH	Hyoid bone	27.6	1.1	

*Width and thickness were measured at the point of attachment

**In addition, TH attached to the oblique line of the thyroid cartilage with single muscle fibers at the level of the distal half of the muscle

In the present case, the TH lay in the carotid triangle and was covered only by the omohyoid muscle. The sternohyoid muscle was located medially to the TH and was partially fused to it. Both heads of this muscle were perfused with the superior thyroid artery. Their nerve branch came from the hypoglossal nerve and was located anteriorly to the omohyoid muscle.

The thyrohyoid muscle was carefully dissected to avoid measurement errors and to accurately identify their positions relative to each other. Measurements were taken twice with up to 0.1 mm accuracy, using an electronic caliper (Mitutoyo Corporation, Kawasaki-shi, Kanagawa, Japan). No additional anatomical variations were recognized in this region.

## Discussion

Branchial arches give rise to lower face, neck, and part of the upper thorax, as well as the muscles in these regions, which are formed from the mesoderm of the arches and neural crest cells between the 4th and 7th week of a gestation [14]. Sato et al. [22] state that most myotomes form in the chest area during the 5th week of gestational life. The infrahyoid, scaleni and prevertebral muscles arise from the myoblast of cervical myotomes [29]. The infrahyoid group is considered as an intermediate grade of development muscles, and it belongs to the group of hypobrachial muscles which phylogenetically are similar to the musculature of the tongue. In their study carried out in fetuses, Muller et al. [16] observed that TH’s were strongly attached to both the body and the greater horn of HB. Additionally, they described the presence of tendinous connections between TH and SH [16].

Infrahyoid muscles are characterized by high variability [15], especially omohyoid [9, 11, 21, 23]. TH is the most constant of these and is relatively less variable in terms of morphology. Its most common variation is fusion with sternothyroid muscle [15]. Mori [15] described the presence of a continuous TH with the ST in 68.2% of the cadavers examined, significantly more often on the right side. The TH we noticed was completely separated from the SH. The literature also reports the occurrence of a very rare additional TH, which is described as the Von Sommering muscle [10]. Regarding its thickness and width, it is greater in males, especially on the right side [3]. The variability of this muscle is generally associated with its origin and insertion point [1].

Usually, TH originates from the oblique line of the thyroid cartilage [25]. Gruber [8] described TH that had its origin in the superior horn of the thyroid cartilage. Among fetuses, attachment to the body of the HB has also been observed [16]. In the available literature, we can also find information about an extremely rare variant of the thyrohyoideus originating only in the cricoid cartilage. This variant of TH is called the cricohyoid muscle [1]. The TH that we identified is a combination of the two variants mentioned above of this muscle. It has two bellies with two separate origins in the thyroid and cricoid cartilages. These two bellies merge in the region of the HB and have a common point of attachment. In view of the fact that the cricohyoid muscle is described as a variant of thyrohyoid, we speculate that this variant may be called the cricothyrohyoid muscle [1].

Among the infrahyoid muscles, the TH has the strongest attachment to the HB, and occurs mainly at the medial two thirds of the lower border of the greater horn [24]. In terms of the attachment part of the TH, based on the overlap of the infrahyoid muscles, Tanaka distinguished three types of insertion [28]. Type 1, in which the TH was covered by the OH and the SH, type 2, in which the TH was covered only by the OH, and type 3, in which the TH was not covered by any of them. In his study, type 2 was the most common [28]. In the following years, Sonoda et al. [24] simplified this classification by distinguishing only two types. The thyrohyoid muscle was not covered by SM and OM as type 1 (corresponding to Type 3 distinguished by Tanaka), and type 2 corresponding to type 1 and 2 proposed by Tanaka). In the work published by Sonoda et al. [24], type 1 was the most common. The TH we identified was covered only by the OH and at the point of insertion (medial two thirds of the greater horn of the HB) it was partially merged with the SH.

Infrahyoid muscles are important landmarks in the prelaryngeal zone [26]. The anatomical positions of the TH and SH are crucial during the surgical approach to the thyroid gland [2]. Moreover, the posterior border of the TH is used as a landmark for the point of penetration through the thyrohyoid membrane (TM) by the superior laryngeal nerve (SLN) [18]. In their research, Paraskevas et al. [18] determined the branching scheme and topography of the internal branch of SLN (iSLN). The iSLN was divided most often into three branches, just before the TM breakthrough. In almost 80% of cases, these branches pierces the TM 10–90 mm away from the posterior border of the TH, and then extend deep into the TH along with the superior laryngeal vessels [18, 27]. This region is called the danger zone, due to the high risk of damage to the iSLN and laryngeal vessels [18]. Moreover, iSLN neuralgia may be the cause of pain accompanied by sensitivity in the lateral cervical zone. In such a case, the posterior margin is used as a landmark for the injection of local nerve blockers. Knowledge of the anatomical variation in this region, especially the landmarks for key structures like iSLN, is essential to avoid intraoperative complications. Lack of knowledge of the potential occurrence of this type of TH can cause a poor estimate of the position of the iSLN and potentially contribute to its damage, if such a variant occurs.

Infrahyoid muscles are successfully used during reconstructions of the pharyngolaryngeal and cervical esophagus as a myocutaneous flap [5]. The infrahyoid muscle flap is indispensable for closing the defect that affects the ventral floor of the oral cavity. However, during this procedure, the TH is left undisturbed to save structures such as laryngeal vessels and the SLN [20, 31]. Peng et al. [20] reported that in medium defects in the neck zone, TH can be detached from thyroid cartilage and relocated to the damaged place. TH can also be considered as a soft palate graft material. In this case, an additional advantage is that innervation can be preserved, which prevents atrophy and translates into better scarring results, even among elderly patients [6, 13]. A thorough knowledge of the morphology of this muscle, the location of its attachment points and its innervation is essential for this type of procedure. The appearance of any variability should be considered.

Two-headed TH potentially offers significantly greater reconstruction options than its standard counterpart. Since this variant of TH has two bellies connected near the attachment point, by attaching this muscle with two free ends of separate heads, we can obtain a much longer muscle graft than its single-belly counterpart. Two heads with separate attachments can be fixed to remote locations, such as two different anatomical structures. Moreover, because of the presence of two bellies, only one of them can be used for a graft. Thus, the graft material is obtained, however, the function of the TH is still preserved. Additionally, we speculate that the presence of a two-headed TH may minimize the risk of complete loss of function of this muscle as a result of its intraoperative damage.

## Conclusions

From a morphological point of view, the thyrohyoid muscle is generally stable. There is little information about its variability in the available literature. Therefore, any findings seem to be of value, particularly for surgeons dealing with the prelaryngeal area. In addition, the two-bellied thyrohyoid muscle may provide more options as a muscular graft.

## Data Availability

No datasets were generated or analysed during the current study.
